# Synergistic antibacterial photodynamic therapy of lysine-porphyrin conjugate and metal ions combination against *Candida albicans* and *Mycobacterium tuberculosis*

**DOI:** 10.3389/fphar.2025.1626193

**Published:** 2025-07-31

**Authors:** Xueming Wang, Zhonghua Qin, Ying Wen, Mingxuan Chi, Lixia Zhang, Junping Wu, Tianjun Liu

**Affiliations:** ^1^ State Key Laboratory of Advanced Medical Materials and Devices, Tianjin Key Laboratory of Biomedical Materials, Institute of Biomedical Engineering, Chinese Academy of Medical Science and Peking Union Medical College, Tianjin, China; ^2^ Tuberculosis Precision Testing Center, Tianjin Haihe Hospital, Tianjin, China

**Keywords:** antibacterial photodynamic therapy, lysine-conjugated porphyrin compound, metal ions, synergistic antibacterial therapy, *Candida albicans*, *Mycobacterium tuberculosis*

## Abstract

**Introduction:**

In previous research, antibacterial photodynamic therapy using lysine-porphyrin conjugate LD_4_ effectively inactivated methicillin-resistant Staphylococcus aureus, Pseudomonas aeruginosa, and Escherichia coli; however, it exhibited limited activity against Candida albicans and Mycobacterium tuberculosis.

**Methods:**

To address this limitation, we developed a synergistic antibacterial strategy by combining LD_4_ with Cu^2+^ or Zn^2+^.

**Results:**

Synergy was confirmed via minimum inhibitory concentration and fractional inhibitory concentration index analyses, demonstrating 16- to 64-fold enhanced antibacterial efficacy compared to LD_4_ alone. Mechanistic studies revealed divergent pathways for LD_4_ + Cu^2+^ and LD_4_ + Zn^2+^: Zn^2+^ increased the reactive oxygen species yield and promoted LD4 uptake by pathogens, while LD_4_ + Cu^2+^ induced oxidative damage to cell walls and membranes in darkness, with light exposure exacerbating structural damage. Cytotoxicity assessments confirmed low toxicity, with >90% survival of normal cells at bactericidal concentrations. Fluorescence and infrared spectroscopy characterized metal-LD_4_ complexes, showing stabilization through interactions between amino and pyrrolic imino groups of LD_4_ and metal ions, which promoted non-radiative transitions and fluorescence quenching. Both combinations caused significant bacterial membrane disruption and growth suppression. Notably, cytotoxicity exhibited a biphasic dose-response linked to metal-LD_4_ complexation-dependent particle size changes.

**Discussion:**

This study elucidated the enhanced antimicrobial mechanisms and safety of LD_4_-metal ion combinations. The findings resolve the limitations of LD_4_ while providing a theoretical framework for developing novel therapies against fungal and mycobacterial infections.

## 1 Introduction

Microbial infections have become a major threat to global public health, particularly due to the dual pressures of nosocomial infections and the rapid emergence and spread of drug-resistant bacteria, which pose unprecedented challenges to human health. According to data from the World Health Organization (2014) and various national health institutions (Collaborators, 2022), key pathogens responsible for widespread epidemics include *Streptococcus pneumoniae* (Mitchell and Mitchell, 2010), *Mycobacterium tuberculosis* (*Mtb*) (Sia and Rengarajan, 2019), methicillin-resistant *Staphylococcus aureus* (Li et al., 2012), and *Escherichia coli* (*E. coli*) (Oordt-Speets et al., 2018). Annually, bacterial infections result in an estimated six to eight million deaths globally (Collaborators, 2017), while fungal infections account for approximately 3.75 million fatalities. Projections indicate that by 2050, these figures could reach 10 million deaths annually (Ikuta et al., 2024; Organization, 2014). Antibiotics function by targeting essential processes for bacterial growth through diverse mechanisms and are indispensable for treating bacterial infections (Organization, 2014). However, the current mainstream antibacterial treatment regimens are characterized by significant limitations (Baran et al., 2023). For instance, despite the ability of antibiotics to achieve efficient bactericidal effects through specific targets, such as penicillin-binding proteins (Zapun et al., 2008) and ribosomes (Wilson, 2014), treatment with these agents is challenged by the rapid evolution of drug resistance, which far outpaces the new drug development cycle (Cook and Wright, 2022). The widespread overuse and misuse of antibiotics (Timmerhuis et al., 2023; Aggarwal et al., 2024), despite decades of rigorous pharmacological investigation, have precipitated a critical public health crisis: accelerated evolution of antibiotic resistance in bacterial and fungal pathogens (Blair et al., 2015). This situation arises primarily from selection pressures driving genetic mutations within microbial populations, which in turn foster rapid pathogen adaptation through horizontal gene transfer and efflux pump upregulation (Ma et al., 2023). Consequently, such adaptive mechanisms diminish the clinical efficacy of existing antimicrobial agents and create a self-reinforcing cycle wherein resistance development outpaces the discovery and deployment of novel antibiotics, thereby threatening the long-term viability of antimicrobial drug pipelines. In resource-constrained settings, social and economic barriers significantly hinder access to effective antibiotics (Iskandar et al., 2021). Moreover, their efficacy is often restricted in treating biofilm-associated chronic infections, including chronic wound infections and implant-related infections (Sharma et al., 2016). Furthermore, metal-based antibacterial materials (Yang et al., 2022), such as silver (Yin et al., 2020), zinc (Riduan and Zhang, 2021), and copper ions (Mitra et al., 2020), have garnered attention for their broad-spectrum antibacterial properties and relatively low potential to induce drug resistance (Ren et al., 2022). Nevertheless, their clinical application is hindered by dose-dependent cytotoxicity and the potential risk of spreading metal resistance genes, such as *silE* (Monneau et al., 2023) and *copA* (Li et al., 2023). Considering these dual constraints—dose-dependent cytotoxicity compromising therapeutic windows and horizontal transfer of resistance genes—novel combinatorial strategies leveraging photodynamic therapy (PDT) show unique promise. Particularly, porphyrin-based photosensitizers like LD4 offer distinct advantages: their innate metal-chelating capacity enables formation of stable complexes that sequester toxic free ions (reducing systemic exposure), while simultaneous ROS generation disrupts bacterial membranes through physical oxidation, circumventing conventional resistance mechanisms (Xu et al., 2016). Therefore, there is an urgent need to develop novel antibacterial strategies to effectively address this challenge. In this context, innovative material designs like Zn-Cu-In-S/ZnS-mTHPP conjugates emerge as promising alternatives. As recently demonstrated, such quantum dot-porphyrin hybrids not only enhance photodynamic tumor cell killing by 72% but also exhibit inherent light-independent antibacterial activity against pathogens including *E. coli*—offering dual therapeutic modalities that bypass traditional cytotoxicity and resistance limitations(Tsolekile et al., 2022).

Antibacterial photodynamic therapy (aPDT) is a novel physical and chemical bacterial inactivation technology based on photosensitizers (Møller et al., 2005). It physically kills bacteria through the burst of reactive oxygen species (ROS) mediated by photosensitizers (Jiang et al., 2024). Its mechanism of action can bypass traditional drug resistance barriers, providing a new solution for overcoming the dormancy of persister cells, biofilm encapsulation, and drug penetration obstacles (Wu et al., 2023). The three key elements of aPDT are photosensitizers, oxygen, and light (Correia et al., 2021). As the core component, the structural differences of photosensitizers determine their therapeutic effects on different disease indications. However, the use of aPDT against certain pathogens is subject to several limitations. These challenges include the risk of systemic toxicity caused by cationic photosensitizers, the potential for light-induced damage to surrounding healthy tissues, the limited light penetration in deep tissues, the decreased effectiveness against persistent bacteria, and the lack of a standardized evaluation framework (Puttaswamy et al., 2023). In our previous studies, we synthesized a series of amino acid-conjugated porphyrins and conducted detailed structural characterizations of these compounds (Meng et al., 2015). A systematic evaluation of their antimicrobial activities was also conducted. We found that compound LD_4_ exhibited promising aPDT effects against methicillin-resistant *Staphylococcus aureus*, *Pseudomonas aeruginosa*, and *E. coli*. The underlying mechanism involves the generation of ROS upon light activation, which induces bacterial oxidative stress responses (Xu et al., 2016). However, LD_4_ exhibits relatively weak antibacterial activity against *Mtb* and *Candida albicans* (*C. albicans*) (minimum inhibitory concentration [MIC] >125 μg/mL), primarily due to the low uptake efficiency of LD_4_ by these pathogens. Notably, in addition to its aPDT effect, LD_4_ significantly enhances the intracellular levels of metal ions such as Cu^2+^ in bacteria. Based on these findings, in this study, we investigated a combination therapeutic strategy involving exogenous supplementation of metal ions (e.g., Cu^2+^, Zn^2+^) synergistically with LD_4_ to broaden the antibacterial spectrum of LD_4_ and address its limited efficacy against certain pathogens. We propose that Cu^2+^/Zn^2+^ enhances LD_4_’s membrane affinity or ROS generation via complexation.

## 2 Materials and methods

### 2.1 Materials

Copper sulfate pentahydrate (CuSO_4_·5H_2_O), zinc acetate [Zn(OAc)_2_], ferric chloride hexahydrate (FeCl_3_·6H_2_O), magnesium Sulfate (MgSO_4_), manganous acetate [Mn(OAc)_2_], chloroform, 3-morpholino-2-propanesulfonic acid, benzylsulfanilimide F, potassium bromide (KBr), disodium ethylenediaminetetraacetate, MeOH, dimethyl formamide, dimethyl sulfoxide (DMSO), 2,7-dichlorofluorescein diacetate (DCFH-DA), and 50% glutaraldehyde were purchased from Bide Pharmaceutical Technology Co., Ltd. (Shanghai, China). Lysozyme (egg white) was purchased from Shanghai Yuanye Bio-Technology Co., Ltd. (Shanghai, China). Cell Counting Kit-8 (CCK-8) was obtained from Biosharp Biotechnology Co., Ltd. (Beijing China). Middlebrook 7H9 broth medium and modified Lowenstein - Jensen medium base were obtained from Signature Biotechnology Co., Ltd. (Guangzhou, China). Sabouraud’s medium was obtained from Beijing Solarbio Technology Co., Ltd. (Beijing, China). LD_4_ was synthesized as previously described (Meng et al., 2015). The standard strain of *Mtb* H37Rv, the drug-sensitive strain 63, 64 and the drug-resistant strain 22,26 (resistant to rifampicin and isoniazid) were provided by the Tianjin Haihe Hospital (Tianjin, China). Ethical approval for the utilization of clinical strains was obtained from the Tianjin Haihe Hospital Medical Ethics Committee (Approval No. 2024HHQX-002). Mouse fibroblasts (3T3), human immortalized keratinocytes (HaCaT), human normal liver cells (LO2), and human bronchial epithelial cells (BEAS-2B) were obtained from Wuhan Pricella Biotechnology Co., Ltd. (Wuhan, China). Semiconductor laser (model 7404, Intense, North Brunswick, NJ, United States) and optical power meter (LM1; Carl Zeiss, Oberkochen, Germany) were used.

### 2.2 Determining MIC and minimum bactericidal concentration, and synergy assessment for metal ions and LD_4_

For *C. albicans* and *Mtb*, the MIC was determined using the broth microdilution method. Serial two-fold dilutions of metal ions solution were prepared in Sabouraud’s or Middlebrook 7H9 broth medium, and LD_4_ was prepared in phosphate-buffered saline, ranging from 1,000–1.95 μg/mL. *C. albicans* strains were grown to the logarithmic phase and then adjusted to a standard inoculum density. The *Mtb* was inoculated onto the modified Löwenstein - Jensen medium and incubated at 37°C in a constant temperature incubator. After approximately 4 weeks of incubation, the culture reached the logarithmic growth phase. Subsequently, the *Mtb* colonies were harvested from the medium by scraping and resuspended in 2 mL of normal saline. Next, the bacterial suspension was sonicated for 120 s to ensure uniform dispersion. A bacterial concentration equivalent to 1 McFarland standard (approximately 3 × 10^8^ CFU/mL) was achieved and subsequently diluted to 10^5^ CFU for further experiments. Aliquots of the bacterial suspension (100 μL) were added to each well of the 96-well microdilution plate containing the metal ions or LD_4_ concentrations (100 μL). For *C. albicans*, the fungal suspension and drug solution were co-incubated for 30 min; in contrast, for *Mtb*, the bacterial suspension and drug solution were co-incubated for 24 h. The plates were exposed to a 650 nm laser with an energy density of 6 J/cm^2^ for 10 min and subsequently incubated at 37°C for either 24 h or 2 weeks, depending on the organism. The MIC was defined as the lowest concentration of the test compound that completely inhibited visible bacterial growth after incubation. The minimum bactericidal concentration was determined following the MIC assay. For *C. albicans* and *Mtb*, suspensions from wells showing no visible growth (i.e., at or above the MIC) were subcultured onto sterile Sabouraud’s agar or Lowenstein - Jensen medium plates, respectively. These plates were then incubated under the same conditions used for the respective MIC assays. The minimum bactericidal concentration was defined as the lowest concentration of the test compound that resulted in no visible growth on the agar plates, indicating a reduction in viable bacteria or fungi by at least 99.9% compared to the untreated control.

The antimicrobial synergistic effects of metal ions and LD_4_ were evaluated against the bacterial strains *C. albicans* and *Mtb* using the Checkerboard dilution method. Serial concentrations of metal ions and LD_4_ were prepared to achieve final concentrations of 4MIC, 2MIC, MIC, 1/2MIC, 1/4MIC, 1/8MIC, 1/16MIC, and 0 in the respective wells of a 96-well microdilution plate.

The fractional inhibitory concentration index (FICI) for each drug in combination was calculated using the following formulas (Barbara et al., 2006):

$$\text{FICI}={\text{FIC}}_{\text{metal}\;\text{ions}}+{\text{FIC}}_{{\text{LD}}_{4}}=\frac{{\text{MIC}}_{\text{metal}\;\text{ions}\;\text{in}\;\text{combination}}}{{\text{MIC}}_{\text{metal}\;\text{ion}\;\text{independently}}}+\frac{{\text{MIC}}_{{\text{LD}}_{4}\;\text{in}\;\text{combination}}}{{\text{MIC}}_{{\text{LD}}_{4}\;\text{independently}}}$$

Synergistic effects were defined as FICI ≤ 0.5, additive effects as 0.5 < FICI ≤ 1, indifferent effects as 1 < FICI ≤ 4, and antagonistic effects as FICI > 4.

### 2.3 Assessment of LD_4_ uptake by *C. albicans* and *Mtb*

To investigate the synergistic mechanism of Cu^2+^/Zn^2+^ and LD_4_ against *C. albicans* and H37Rv, the intracellular uptake of LD_4_ was quantified using flow cytometry. H37Rv was cultured on Löwenstein - Jensen medium at 37°C until the logarithmic growth phase. Bacterial colonies were harvested using sterile loops, suspended in normal saline, and homogenized by ultrasonication (5 s pulse/5 s pause, 12 cycles, 80% amplitude) to obtain a uniform suspension (5 McFarland standard). *C. albicans* was cultured on Sabouraud’s medium at 28°C until the logarithmic growth phase, and the suspension was concentrated to 5 McFarland standard. Three experimental groups were established: LD_4_ alone (31.25 μg/mL); LD_4_ (31.25 μg/mL) + Cu^2+^; and LD_4_ (31.25 μg/mL) + Zn^2+^. Metal ion concentrations ranged from 3.13 to 50 μg/mL (two-fold serial dilution). For each condition, 1 mL of bacterial suspension was mixed with 500 μL of LD_4_ (125 μg/mL stock solution) and 500 μL of metal ion solution in sterile tubes. After incubation for 24 h in the dark at 37°C, unbound LD_4_ was removed by centrifugation (4,000 × *g*, 10 min, 4°C), and the bacteria were subsequently washed thrice with saline (0.9% NaCl, Signature Biotechnology Co., Ltd. ,Guangzhou, China). Washed bacteria were resuspended in 1 mL saline, filtered through a 40 μm cell strainer, and analyzed using a NovoCyte Quanteon flow cytometer (Agilent Technologies Inc. ,City of Santa Clara, CA, United States). PE-Cy7 channel (excitation/emission: 488/780 nm) was selected based on LD_4_ fluorescence. Data from 100,000 events per sample were acquired at high flow rate (66 μL/min), with unstained bacteria serving as negative controls. Fluorescence compensation was applied using single-stained LD_4_ samples. Three independent replicate experiments were conducted for each group.

### 2.4 Determination of fluorescence and infrared spectra for metal ions in combination with LD_4_

A stock solution of LD_4_ (10 mg/mL) was prepared in DMSO. Metal ion stock solutions (Zn^2+^ and Cu^2+^) were prepared at concentration of 2,000 ppm (weight/volume) in ultrapure water. Three experimental groups were designed to evaluate quenching effects: Control group with LD_4_ alone (31.25 μg/mL); LD_4_ + metal ions (31.25 μg/mL LD_4_ + 50 μg/mL combined Zn^2+^ or Cu^2+^). Aliquots of diluted metal ions (500 μL) and LD_4_ working solution (500 μL) were mixed in 2 mL microcentrifuge tubes, followed by incubation at 25°C for 10 min. Temperature-dependent experiments were conducted at 20°C, 10°C, and 0°C using a thermostatically controlled ethanol bath. Fluorescence spectra were acquired using a fluorescence spectrophotometer (Hitachi F4600, Japan) with excitation at 390 nm and emission scanning from 400 to 800 nm. Slit widths were set at 2 nm for both excitation and emission. The Stern - Volmer equation was applied to quantify quenching efficiency:

$$\frac{I_{0}}{I}=1+K_{q}\tau_{0}\left[Q\right]$$

where *I*_0_ and *I* represent fluorescence intensities without and with quencher, respectively; *Kq* is the Stern - Volmer quenching constant; *τ*_0_ denotes the intrinsic fluorescence lifetime; and [*Q*] indicates quencher concentration. The quenching efficiency (*η*) was calculated using the following formula:

$$\eta =\frac{{FI}_{{LD}_{4}}-{FI}_{{LD}_{4}+ion}}{{FI}_{{LD}_{4}}}\times 100\%$$

where *FI*_*LD*4_ and *FI*_*LD4+ion*_ correspond to maximum fluorescence intensities of LD_4_ in the absence and presence of metal ions, respectively.

Infrared spectra were acquired using a Thermo Fisher Scientific Nicolet iS20 Fourier-transform infrared (FTIR) spectrometer (Waltham, MA, United States). LD_4_ samples were analyzed in three states: LD_4_ alone; LD_4_-Cu^2+^ complex; and LD_4_-Zn^2+^ complex. The concentrations of LD_4_, Cu^2+^/Zn^2+^ were 31.25 μg/mL and 50 μg/mL, respectively. The complex was prepared via freeze-drying. Each sample (5 mg) was homogeneously mixed with anhydrous KBr (spectroscopic grade, 500 mg) using a mortar and pestle for 15 min under ambient conditions. The mixture was pressed into transparent pellets (10 mm diameter) under 10 MPa pressure for 35 s using a hydraulic pellet press. Pellets were mounted in the sample compartment, and background spectra were collected from clean KBr crystals. FTIR measurements were performed over the wavenumber range of 400–4,000 cm^−1^ with a spectral resolution of 4 cm^−1^. Each spectrum represented the average of three independent scans to ensure reproducibility.

### 2.5 Determination of ROS yield

DCFH-DA was employed as the fluorescent probe for quantifying ROS production. The DCFH-DA powder was dissolved in DMSO to prepare a 10 mM stock solution, which was aliquoted and stored at −20°C in the dark. During the experiment, the stock solution was diluted 1:1,000 prior to use. LD_4_ was used at a concentration of 31.25 μg/mL, while Cu^2+^ and Zn^2+^ metal ions were combined with LD_4_ at concentrations of 50, 25, 12.5, 6.25, and 3.13 μg/mL. For each experimental group, 1 mL of solution was prepared, and 1 μL of the DCFH-DA stock solution was added. After thorough mixing, the samples were exposed to a 650 nm laser with a light density of 6 J/cm^2^ for 10 min. Following irradiation, the samples were diluted three-fold, and their fluorescence intensities were measured using a fluorescence spectrometer. Change in the rate of ROS production was calculated using the following formula:

$$R_{\Phi_{\Delta }}=\frac{F_{s}\times \eta_{s}^{2}}{F_{r}\times \eta_{r}^{2}}\times 100\%$$

*F*_*s*_ and *F*_*r*_ represent areas under the fluorescence curve of the sample and LD_4_ control groups, respectively, at 525 nm; *η*_*s*_ is the refractive index of the sample solution, and *η*_*r*_ is the refractive index of the solvent in the LD_4_ control group.

### 2.6 Transmission electron microscopy (TEM) analysis

The dispersion of *Mtb* was performed as described in Section 2.2, and a bacterial suspension with a concentration of 10 MCF was prepared. Subsequently, the suspension was transferred to a 5 mL centrifuge tube and centrifuged at 4,000 rpm for 10 min. The supernatant was discarded. The stock solutions of Cu^2+^ and Zn^2+^ were diluted to a final concentration of 31.25 μg/mL and added to the treated bacterial suspension. The mixture was incubated at 37°C for 24 h. After incubation, the samples were centrifuged again at 4,000 rpm for 10 min, the supernatant was discarded, and the pellets were washed thrice with 1 mL of physiological saline under identical centrifugation conditions. Following washing, 5% glutaraldehyde solution (2 mL) was added to each sample for fixation. The samples were thoroughly mixed and fixed at 4°C for 72 h. Next, the fixed samples were observed under a TEM (FEI Tecnai F20, Hillsboro, OR, United States) for morphological analysis and image acquisition. Elemental mapping signals were subsequently collected to determine the distribution characteristics of Cu^2+^ and Zn^2+^ within the bacteria.

### 2.7 Cytotoxicity of the combination of metal ions and LD_4_

Cytotoxicity was assessed using the CCK-8 assay in various cell lines, including HaCaT, 3T3, BEAS-2B, and LO2. Concentrations for Cu^2+^ and Zn^2+^ monotherapy were set at 6.25, 12.5, 25, 50, and 100 μg/mL, while those for Cu^2+^/Zn^2+^-LD_4_ were set at 7.81 + 3.13, 15.63 + 6.25, 31.25 + 12.5, 62.5 + 25, and 125 + 50 μg/mL based on the FIC results. After the cells reached the logarithmic growth phase, they were co-incubated with the respective treatment at 37°C for 30 min and subsequently irradiated with a 650 nm laser (energy density: 6 J/cm^2^) for 10 min. Thereafter, cells were transferred to a cell incubator and incubated for an additional 24 h. Following incubation, enhanced CCK-8 dilution solution (10 μL) was added to each well of both the experimental and control groups. The plates were then incubated at 37°C and 5% CO_2_ for 30 min. Following incubation, the plates were removed, and the absorbance at 450 nm was measured for each well using an enzyme-linked spectrophotometer (Thermo Fisher Scientific, VARIOSKAN FLASH, Waltham, MA, United States).

### 2.8 Determination of particle size of the combination of LD_4_ and metal ion

The changes in particle size upon mixing LD_4_ solution with Cu^2+^ and Zn^2+^ at specific concentrations were determined using a laser particle size analyzer (Malvern Panalytical ZETASIZER Ultra ZSU3305, Malvern, United Kingdom). Three mixed solutions were prepared with concentrations of 125 + 50, 62.5 + 25, and 31.25 + 12.5 μg/mL (LD_4_ + Cu^2+^/Zn^2+^), respectively. The solutions were treated with ultrasound for 15 min to ensure homogeneity. Subsequently, the particle size changes of the mixed solutions were measured at 25°C. Prior to each measurement, the instrument was stabilized for 2 min to ensure measurement accuracy.

### 2.9 Statistical analysis

Data processing was performed using GraphPad Prism software (version 8.0.1). All experiments in this chapter were independently repeated three times. Statistical analyses were carried out using GraphPad Prism 8.0.2 software, and image processing as well as data export were conducted using ImageJ software. Results are presented as mean ± standard deviation (mean ± SD). Each variable was analyzed using one-way ANOVA, and the significance of differences between groups was assessed via Tukey’s multiple comparison test (p < 0.05).

## 3 Results and discussion

### 3.1 Synergy assessment for metal ions and LD_4_

The MIC values of each metal ion and LD_4_ against *C. albicans* alone were determined using the broth microdilution method, providing a basis for investigating the synergistic effects between metal ions and LD_4_. Significant antifungal activity of Cu^2+^, Zn^2+^, Fe^3+^, and Mn^2+^ against *C. albicans* was demonstrated, with MIC values of 40 μg/mL. In contrast, Mg^2+^ exhibited weaker antifungal efficacy, with an MIC value of 80 μg/mL. Additionally, LD_4_ showed limited antifungal activity against *C. albicans*, with an MIC value of 125 μg/mL. Based on the above results, we observed that the antifungal activity of certain metal ions (Cu^2+^, Zn^2+^, Fe^3+^, and Mn^2+^) was significantly higher than that of LD_4_. As a lysine-conjugated amino phenyl porphyrin photosensitizer, LD_4_ had been suggested by previous studies to potentially exert synergistic effects through its ability to complex metal ions (Cu^2+^) and accumulate in pathogenic microorganisms (manuscript in preparation). Based on these findings, we propose that the antifungal activity of LD_4_ against *C. albicans* can be enhanced through the addition of supplementary metal ions. To verify this hypothesis, the checkerboard dilution method was used to quantitatively assessed the synergistic effect of metal ions combined with LD_4_ against *C. albicans*.

**FIGURE 1:**
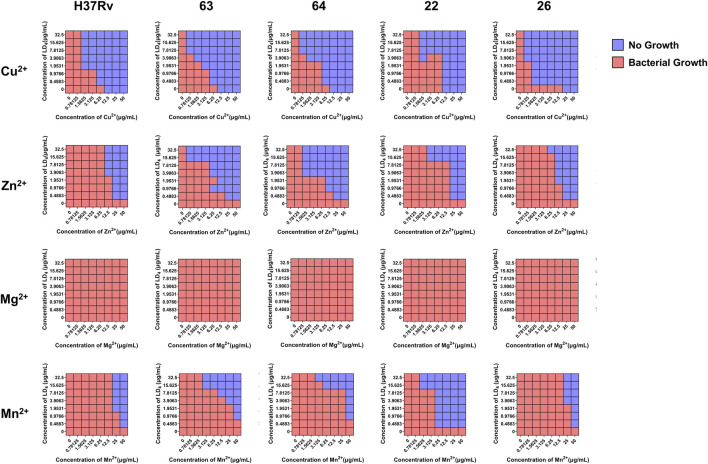
The synergistic effect of LD_4_ in combined with metal ions against *Mycobacterium tuberculosis* (*Mtb*) (H37Rv, 63, 64, 22, 26). Red and blue wells indicate the presence and absence of bacterial growth, respectively.

As shown in Table 1, the metal ions with the most effective synergistic effects were Cu^2+^, Zn^2+^, Mn^2+^, and Mg^2+^, all of which had FICI values < 0.5. Compared to their individual utilization, the MIC of these four metal ions decreased by 16- to 32-fold, while the MIC of LD_4_ decreased by 64- to 16-fold. These results demonstrated that when combined with LD_4_, Cu^2+^, Zn^2+^, Mn^2+^, and Mg^2+^ exhibited a significant synergistic antibacterial effect against *C. albicans*. The FICI value of Fe^3+^ was 0.75, indicating an additive effect, which was higher than those of other tested metal ions. Consequently, Cu^2+^, Zn^2+^, Mn^2+^, and Mg^2+^, which exhibited synergistic effects, were selected for further investigation of the synergistic antibacterial activity against *Mtb* with LD_4_. We selected one standard *Mtb* strain, two sensitive strains, and two drug-resistant strains (resistant to rifampicin and isoniazid) for this study. The results are illustrated as a heatmap (Figure 1), while the corresponding FIC and FICI values are summarized in Table 2. The results demonstrated that Mg^2+^ exhibited no significant synergistic effect against *Mtb*, with an MIC value exceeding 50 μg/mL. Furthermore, no synergistic effect was observed when Mg^2+^ was combined with LD_4_. In contrast, the combination of LD_4_ with Cu^2+^, Zn^2+^, or Mn^2+^ resulted in pronounced growth inhibition of *Mtb*. Quantitative analysis demonstrated that the synergistic antibacterial effects of metal ions combined with LD_4_ were more pronounced against sensitive strains and the standard strain H37Rv than resistant strains. The FIC values for these strains were lower than those for drug-resistant *Mtb*, suggesting that bacterial growth could be effectively inhibited at reduced drug combination concentrations (Eumkeb and Chukrathok, 2013). These three metal ions exhibited synergistic effects with LD_4_, with FICI values consistently <0.5. Notably, Cu^2+^ and Zn^2+^ demonstrated the most potent synergistic efficacy against *Mtb* with LD_4_. The MIC values of these metal ions were reduced by 8- to 128-fold, while the MIC of LD_4_ decreased by 8- to 64-fold. Furthermore, their FICI values were all < 0.2, indicating a strong synergistic interaction. However, Mn^2+^ exhibited weaker synergistic effect compared to Cu^2+^ and Zn^2+^, although it still demonstrated synergistic efficacy. FIC value of Mn^2+^ (25 μg/mL) was higher than that of Cu^2+^ (0.78–1.56 μg/mL) and Zn^2+^ (0.78–12.5 μg/mL), and there may be a potential risk of manganese ion accumulation toxicity (Rodichkin and Guilarte, 2022). The concentration-dependent antibacterial activity of the LD_4_-Cu^2+^ complex highlights a critical mechanistic subtlety. Specifically, complete inhibition of *Mtb*. Growth was observed exclusively at the synergistic ratio of 3.91 μg/mL LD_4_ and 1.56 μg/mL Cu^2+^ (Figure 1, Row 1-Panel 4 and Row 2-Panel 2). Conversely, deviations from this optimal Cu^2+^ concentration paradoxically restored bacterial viability. This phenomenon is likely attributable to the dual interaction between the stoichiometric chelation of LD_4_ and Cu^2+^ and the pathogen’s intrinsic metal stress adaptation mechanisms. At the defined ratio, LD_4_ and Cu^2+^ may form a stable antimicrobial complex that disrupts membrane integrity or metabolic pathways via targeted interactions. However, concentrations of Cu^2+^ could activate bacterial copper detoxification systems, such as the CtpV efflux pump (a P-type ATPase essential for copper homeostasis in *Mtb.*), thereby diminishing the efficacy of LD_4_. These adaptive responses are consistent with prior studies demonstrating mycobacterial resilience to metal stress through regulated efflux and redox balancing mechanisms (Ward et al., 2008). Based on these findings, Cu^2+^ and Zn^2+^ were selected for subsequent studies on mechanisms and cytotoxicity in combination with LD_4_.

**TABLE 1:** MIC, FIC, and FICI values of metal ions and LD_4_ against *Candida albicans*.

Group	MIC (μg/mL)		FIC (μg/mL)	FICI	Effect
	Metal ion	LD_4_	Metal ion + LD_4_		
Cu^2+^	40	125	2.5 + 1.95	0.078	Synergy
Zn^2+^	40	125	2.5 + 0.98	0.070	Synergy
Fe^3+^	40	125	10 + 62.5	0.75	Additive
Mn^2+^	40	125	2.5 + 3.91	0.16	Synergy
Mg^2+^	80	125	2.5 + 15.63	0.094	Synergy

Effect represents the synergistic situation between metal ions and LD_4_.

Abbreviations: FIC, fractional inhibitory concentration; FICI, fractional inhibitory concentration index; MIC, minimum inhibitory concentration.

**TABLE 2:** MIC, FIC, and FICI values of metal ions and LD_4_ against *Mtb* (H37Rv, 63, 64, 22, 26).

Strain	Group	MIC (μg/mL)		FIC (μg/mL)	FICI	Effect
		Metal ion	LD_4_	Metal ion + LD_4_		
H37Rv	Cu^2+^	12.5	>125	1.56 + 1.95	<0.14	Synergy
	Zn^2+^	100		12.5 + 3.91	<0.16	Synergy
	Mn^2+^	>100		25 + 1.95	<0.27	Synergy
	Mg^2+^	>100		—	—	—
63	Cu^2+^	12.5	>125	0.78 + 7.81	<0.13	Synergy
	Zn^2+^	100		0.78 + 15.63	<0.13	Synergy
	Mn^2+^	>100		3.13 + 15.63	<0.16	Synergy
	Mg^2+^	>100		—	—	—
64	Cu^2+^	12.5	>125	1.56 + 3.91	<0.16	Synergy
	Zn^2+^	100		1.56 + 3.91	<0.05	Synergy
	Mn^2+^	>100		6.25 + 15.63	<0.19	Synergy
	Mg^2+^	>100		—	—	—
22	Cu^2+^	12.5	>125	1.56 + 7.81	<0.19	Synergy
	Zn^2+^	100		3.13 + 15.63	<0.16	Synergy
	Mn^2+^	>100		6.25 + 0.49	<0.07	Synergy
	Mg^2+^	>100		—	—	
26	Cu^2+^	12.5	>125	1.56 + 0.49	<0.13	Synergy
	Zn^2+^	100		12.5 + 1.95	<0.16	Synergy
	Mn^2+^	>100		25 + 3.91	<0.28	Synergy
	Mg^2+^	>100		—	—	—

Effect represents the synergistic situation between metal ions and LD_4_.

Abbreviations: FIC, fractional inhibitory concentration; FICI, fractional inhibitory concentration index; MIC, minimum inhibitory concentration; *Mtb*, *Mycobacterium tuberculosis*.

### 3.2 Uptake of LD_4_ in *C. albicans* and *Mtb*

**FIGURE 2:**
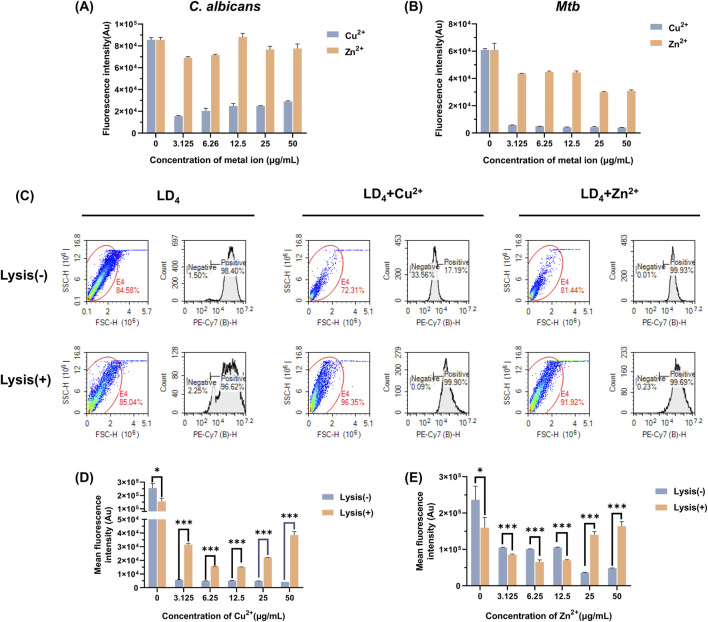
Measurement of LD_4_ uptake by *Mtb* and *C. albicans* under the co-culture conditions of Cu^2+^/Zn^2+^. LD_4_ uptake measurement in **(A)** *C. albicans* and **(B)** *Mtb*. **(C)** Flow cytometry scatter plots and histograms showing LD_4_ uptake by H37Rv under treatment with LD_4_, LD_4_ + Cu^2+^, and LD_4_ + Zn^2+^. LD_4_ binding levels in H37Rv cells and their contents before and after lysis in the **(D)** LD_4_ + Cu^2+^ group and **(E)** LD_4_ + Zn^2+^ group. Lysis(−) and Lysis(+) indicate unlysed and lysed bacterial groups, respectively. *: p < 0.05; **: p < 0.01; ***: p < 0.001. Abbreviations: *C. albicans*, *Candida albicans*; *Mtb*, *Mycobacterium tuberculosis*.

The uptake of LD_4_ by *C. albicans* and *Mtb* was quantitatively analyzed using flow cytometry, with fluorescence intensity serving as the indicator (Setiawati et al., 2017). The results are presented in Figures 2A,B. Compared to the LD_4_ alone group, the fluorescence intensity in the LD_4_+Cu^2+^ group was significantly reduced (p < 0.05). Although the fluorescence intensity in the LD_4_+Zn^2+^ group was higher than that in the LD_4_+Cu^2+^ group, no significant difference was observed compared to the LD_4_ alone group (p > 0.05). Notably, although Cu^2+^ and Zn^2+^ significantly enhanced the antibacterial efficacy of LD_4_ against *C. albicans* and *Mtb*, this improvement did not correlate with drug uptake. The possible reason for this observation is the presence of a complexation-dissociation equilibrium between metal ions and LD_4_ (Tabata et al., 1996). Consequently, the system comprises at least three components, namely, metal ions, LD_4_, and their complexes. These constituents exhibit distinct modes of bacterial interaction, resulting in different biological outcomes. LD_4_ initially binds to the cell wall and partial cytoplasmic membrane of *Mtb*. It disrupts copper transport proteins by photodynamic action, thereby facilitating massive influx of copper ions into the bacterial cells. This process culminates in intracellular copper overload, exerting toxicity and achieving bactericidal effects. Meanwhile, the excessive accumulation of copper ions disrupts bacterial homeostasis significantly, potentially leading to bacterial death. LD_4_ enters bacterial cells via passive diffusion at a relatively low concentration. LD_4_ may bind to the DNA of *Mtb*, disrupting protein synthesis through photodynamic action, which in turn suppresses replication and inhibits bacterial growth. Copper ions may complex with the LD_4_ porphyrin ring, leading to fluorescence quenching of LD_4_ and promoting its aggregation. Consequently, lower fluorescence intensity of LD_4_ is observed in flow cytometry analysis.

To further investigate the underlying causes of this phenomenon, we lysed *Mtb* that had been treated with drugs and analyzed the resulting data using flow cytometry. The scatter plots, histograms, and the fluorescence intensities before and after lysis, are presented in Figures 2C–E. Significant differences were observed in the bacterial scatter plots before and after lysis. The scatter points predominantly shifted toward smaller volume and increased surface roughness, confirming the success of the lysis process (Gienger et al., 2019). Notably, bacterial morphology changes in the LD_4_ combined with Cu^2+^ and Zn^2+^ group were more pronounced compared to the LD_4_ group, indicating a greater lysis effect. These findings suggest that the synergy between metal ions and LD_4_ exerts a substantial impact on bacterial homeostasis, reducing their ability to resist external damage and rendering them more susceptible to lysis. After bacterial lysis, the supernatants were colorless, suggesting that most of the LD_4_ was bound to the cell wall, cell membrane, and intracellular components of *Mtb*; only a small proportion of LD_4_ remained in a free and unbound state. Analysis of lysed *Mtb* following treatment with LD_4_ alone showed a greater release of cellular components but reduced average fluorescence intensity. This evidence indicates that, compared to the other two groups, less LD_4_ was bound to the bacterial contents. In contrast, in the LD_4_ + Cu^2+^ group, lysis resulted in both an increased proportion of cellular contents and a significantly elevated average fluorescence intensity. These findings suggest that under metal ion co-catalysis conditions, *Mtb* exhibits enhanced LD_4_ uptake capability and effectively transports LD_4_ from the cell wall into the bacterial interior, thereby achieving efficient photodynamic antibacterial effects. When the concentration of metal ions reached 50 μg/mL, the fluorescence intensity peaked, which may be closely associated with the transport mechanisms of *Mtb*. In the Zn^2+^ group, a significant difference in LD_4_ uptake was observed between pre- and post-lysis conditions. Under high-concentration conditions (25 μg/mL and 50 μg/mL), the average fluorescence intensity of LD_4_ was significantly increased, indicating that leaked bacterial contents effectively bound to LD_4_, enabling the exertion of the aPDT effect within bacteria. Conversely, under low-concentration conditions (3.13, 6.25, and 12.5 μg/mL), the LD_4_ content decreased after lysis, suggesting that the binding capacity of bacterial contents to LD_4_ was lower compared to that recorded under high-concentration conditions. Additionally, in the LD_4_ group, the fluorescence intensity was significantly decreased, further supporting the aforementioned findings. The experimental results demonstrated that the synergistic interaction of Cu^2+^/Zn^2+^ with LD_4_ enhances the uptake of LD_4_ by *Mtb*, thereby significantly improving its antibacterial efficacy.

### 3.3 Fluorescence and infrared spectra for metal ions in combination with LD_4_

**FIGURE 3:**
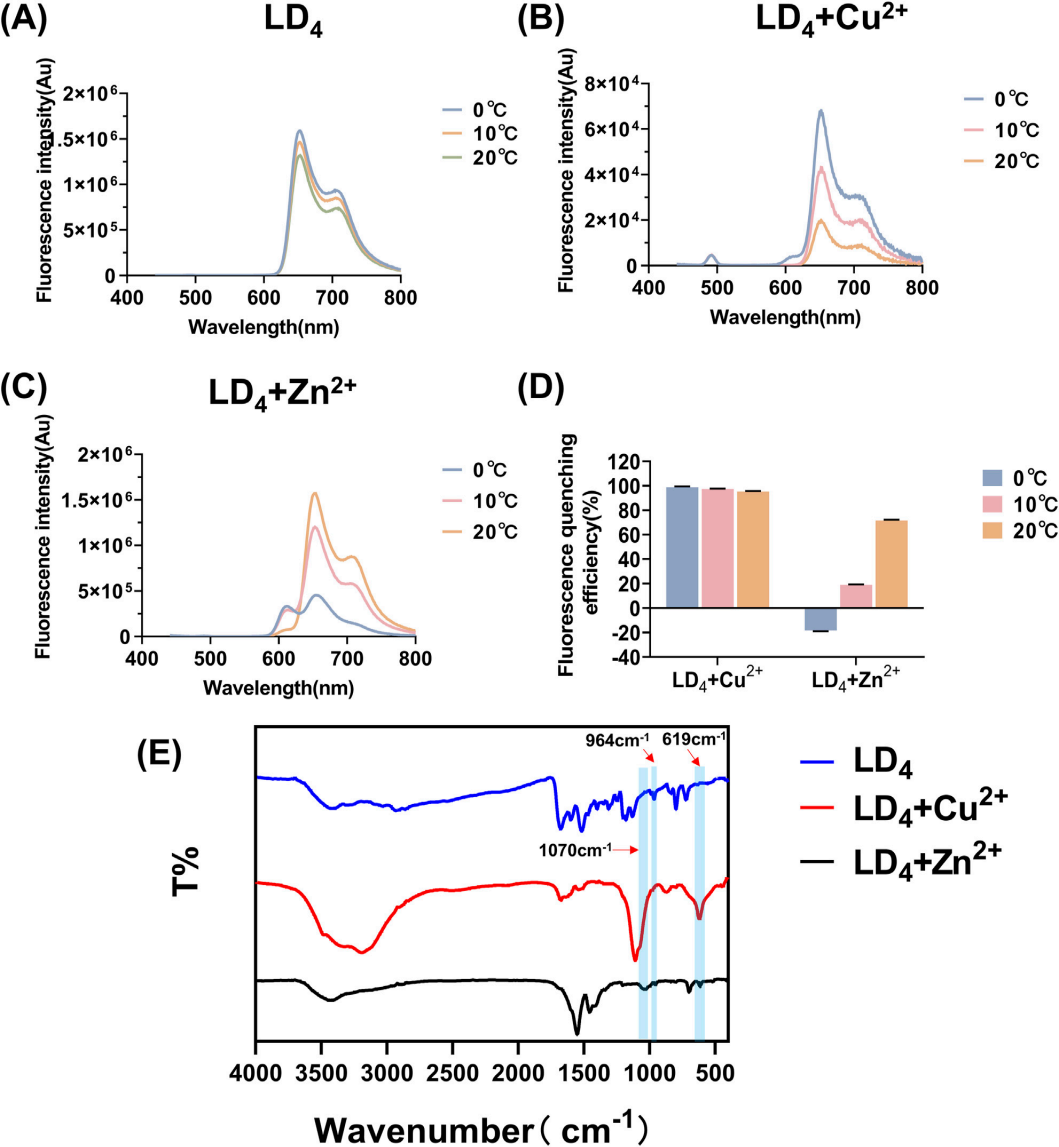
Fluorescence spectra of LD_4_ **(A)**, LD_4_ + Cu^2+^ **(B)**, and LD_4_ + Zn^2+^ **(C)** under various temperature conditions, fluorescence quenching efficiency analysis **(D)**, and infrared spectroscopy measurements **(E)**.

Based on the above findings, we systematically investigated the spectral properties of the complexes formed between metal ions (Cu^2+^ and Zn^2+^) and LD_4_ by analyzing their fluorescence and infrared spectra at various temperatures following combined administration. The corresponding results are presented in Figures 3A–D. Both Zn^2+^ and Cu^2+^ induced fluorescence quenching of LD_4_ under varying temperature conditions. Notably, Cu^2+^ exhibited the most pronounced quenching effect on LD_4_ (Wu et al., 2020). At 20°C, the fluorescence quenching efficiency (η) was >95%. With decreasing temperature, the fluorescence quenching effect progressively intensified, reaching >98% at 0°C. At 0°C, Zn^2+^ induced an enhancement in the fluorescence intensity of LD_4_. With increasing temperature, the η progressively increased from 18.148% to 71.628%. The fluorescence intensity of the LD_4_ + Cu^2+^ group decreased as temperature increased, from 98.823% to 95.437%. In the LD_4_ + Zn^2+^ group, the fluorescence intensity at 650 nm increased with rising temperature, whereas the fluorescence intensity at 612 nm decreased with increasing temperature. This observation is primarily attributed to Cu^2+^ being a tetrahedral metal ion with four coordination sites, which readily achieves coordination saturation. The resulting compounds or polymer structures exhibit stability, and the binding constant increases. Consequently, its overall structure, irrespective of its form, resembles that of a well-defined compound. Thus, its fluorescence behavior as a function of temperature can be explained by traditional molecular principles: as the temperature decreases, the fluorescence intensity increases, leading to highly efficient fluorescence quenching. Zn^2+^ can adopt a pentacoordinate state. The LD_4_ molecule contains four lysine side chains, each providing two amino groups as potential ligands. Consequently, during the binding process, LD_4_ tends to form a tetrahedral coordination structure, which cannot fully satisfy the pentacoordination requirement of Zn^2+^ (He et al., 2011). In the pentacoordinate state, Zn^2+^ exhibits an unsaturated coordination environment, and its structure achieves partial stability through dynamic adjustments. However, this relatively unstable configuration is prone to physical energy-dissipating processes, such as rotation, vibration, and oscillatory motions, which adversely affect fluorescence emission efficiency. When the temperature increases, the complex of LD_4_ with zinc ions facilitates increased molecular collisions and structural reorganization, thereby transitioning the system from an unstable state to a more stable configuration. This reduction in instability leads to decreased internal energy dissipation within the molecules, which enhances fluorescence emission. Conversely, when the temperature decreases, the coordination-unsaturated state induces continuous internal mechanical adjustments within the molecules. These adjustments result in greater vibrational and oscillatory energy losses, which diminish luminescence efficiency. In conclusion, the response of the unstable structural state caused by coordination desaturation to temperature changes is opposite to that of stable structure molecules: when the temperature rises, the fluorescence signal intensifies; while when the temperature drops, the fluorescence intensity weakens.

Furthermore, the infrared spectra of LD_4_, LD_4_ + Cu^2+^, and LD_4_ + Zn^2+^ were recorded to investigate the changes in their functional groups in response to metal ion coordination. The results are presented in Figure 3E. The absorption peaks observed at 964 cm^−1^ correspond to the stretching vibration of -NH- in the inner ring of porphyrin; the peaks at 1,440 cm^−1^ and 1,516 cm^−1^ belong to the deformation vibration of -CH_2_ on the porphyrin ring and the stretching vibration of -C=C-, respectively; the absorption peaks at 1,674 cm^−1^ and 1,598 cm^−1^ correspond to the deformation vibration of -C=O and -NH- in the amide bond. Moreover, the absorption peak at 797 cm^−1^ indicated the presence of a benzene ring. In the LD_4_ + Cu^2+^ group, the disappearance of the absorption peak at 964 cm^−1^ indicated the suppression of -NH- stretching vibrations. The peak at 1,107 cm^−1^ was attributed to the stretching vibration of -S=O, while the peak at 619 cm^−1^ corresponds to the bending vibration of -NH- induced by the coordination of Cu^2+^ with the amino group (Zhao et al., 2019). These results confirmed successful coordination between Cu^2+^ and the free amino groups in the porphyrin ring. Additionally, a broad peak observed in the 3,000–3,500 cm^−1^ region suggests the presence of hydrogen bonding and possible polymer formation. The peak at 3,192 cm^−1^ is associated with the -NH- vibration of secondary amine groups, whereas the peak at 1,070 cm^−1^ may represent the characteristic oxidation state band of metal porphyrins formed upon coordination of Cu^2+^ with the -NH- group in the inner porphyrin ring. The infrared spectroscopic analysis of the LD_4_ + Zn^2+^ group revealed that the absorption peak at 697 cm^−1^ was attributed to the deformation vibration of -NH- induced by the coordination of Zn^2+^ with -NH_2_. The sharp peak at 1,549 cm^−1^ showed a significantly increased intensity and reduced transmittance compared to the peak at 1,598 cm^−1^ in the LD_4_ spectrum. This finding confirmed the formation of N-Zn coordination bonds due to chemical bond changes on the amino group. The peak at 1,454 cm^−1^ corresponded to the symmetric stretching vibration of COO^−^. Moreover, the higher transmittance of the peak at 3,427 cm^−1^ compared to the peak at 3,419 cm^−1^ in the LD_4_ spectrum indicated a weakened stretching vibration of the amino group (Kurtikyan et al., 2014). This evidence suggested partial participation of amino groups in Zn^2+^ coordination, while some amino groups remained uncoordinated.

### 3.4 ROS yield of the combination of metal ions and LD_4_

**FIGURE 4:**
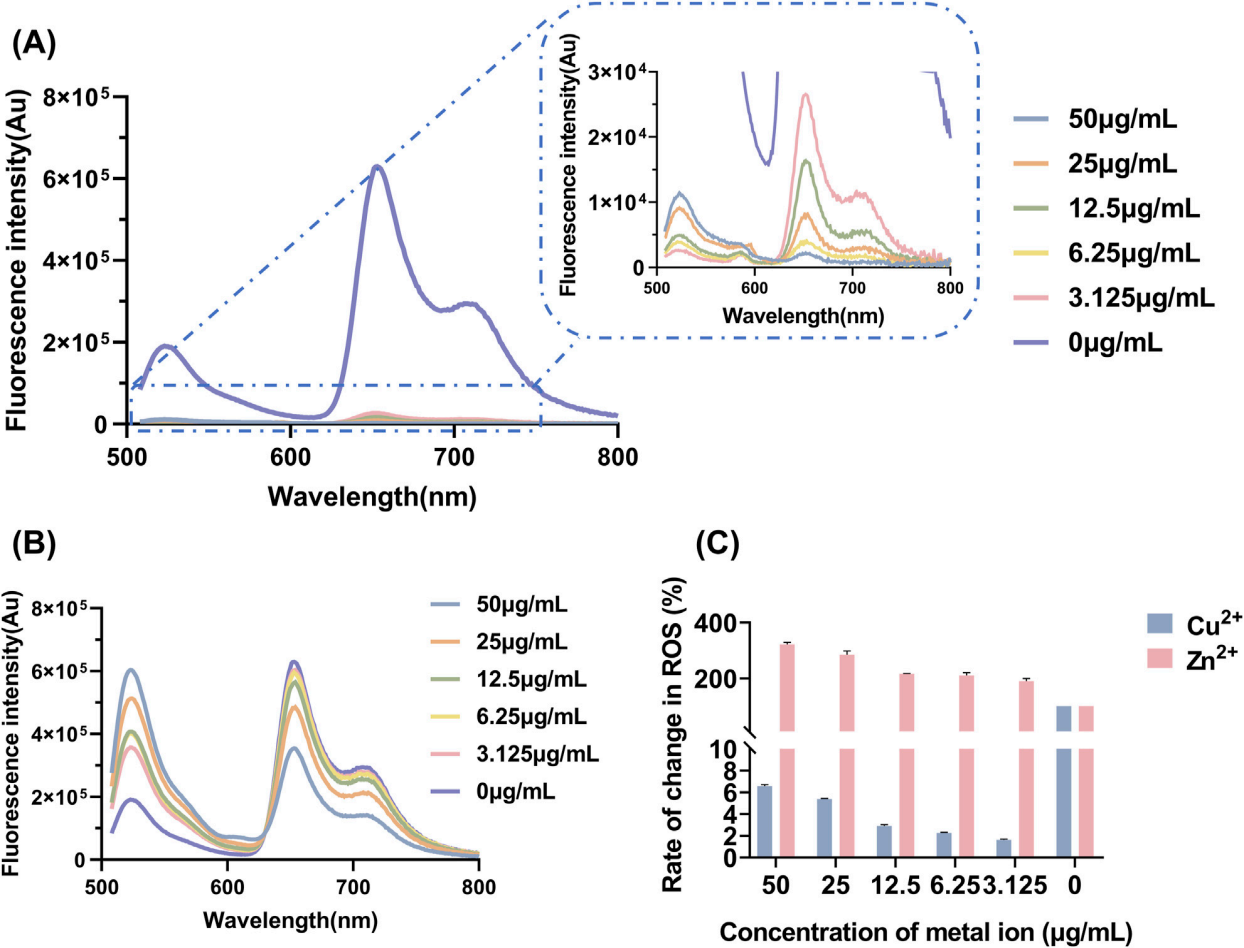
Yield and changes in the levels of ROS after LD_4_ was combined with varying concentrations of metal ions. Fluorescence spectra of DCFH-DA upon combination of LD_4_ with different concentrations of **(A)** Cu^2+^ and **(B)** Zn^2+^. **(C)** Changes in the levels of ROS after LD_4_ was combined with metal ions. Abbreviations: *C. albicans*, *Candida albicans*; DCFH-DA, 2,7-dichlorofluorescein diacetate; *Mtb*, *Mycobacterium tuberculosis*; ROS, reactive oxygen species.

The determination of ROS yield serves as a critical indicator for evaluating the oxidative damage exerted by antibacterial agents on pathogen cells (Khorsandi et al., 2022). Measuring ROS yield aids in elucidating the antibacterial mechanism of aPDT, and directly reflects its therapeutic efficacy. The fluorescence spectra of DCFH-DA and the corresponding ROS yield results are presented in Figure 4. As the concentration of Zn^2+^ increased, the production of ROS after LD_4_ irradiation gradually increased. In contrast, with increasing concentrations of Cu^2+^, the ability to generate ROS progressively diminished. Cu^2+^ induced a fluorescence quenching effect on LD_4_, leading to a significantly reduced ROS yield rate compared to LD_4_ alone. At a Cu^2+^ concentration of 50 μg/mL, the ROS production rate decreased to only 6.58% ± 0.13% of that observed with LD_4_ alone. Conversely, the addition of Zn^2+^ significantly enhanced the ability of LD_4_ to generate ROS, achieving an ROS production rate of 322.35% ± 7.30% at a Zn^2+^ concentration of 50 μg/mL. These experimental results demonstrated that the synergistic interaction between Zn^2+^ and LD_4_ significantly increased the ROS yield, enabling efficient bacterial eradication. Although the combination of Cu^2+^ and LD_4_ generated less ROS, it still exhibited notable antibacterial efficacy. The relatively low ROS yield can be attributed to the formation of stable complexes between Cu^2+^ and the amino groups on the side chains of LD_4_. This interaction reduces non-radiative transitions while enhancing radiative transitions, thereby minimizing vibrational and rotational energy losses to the greatest extent (Kejík et al., 2021). The significant antibacterial activity observed may result from the ability of the Cu^2+^-LD_4_ complex to promote the oxidation of bacterial cell wall lipids, which severely compromises the structural integrity of the bacterial cell wall.

### 3.5 TEM analysis

**FIGURE 5:**
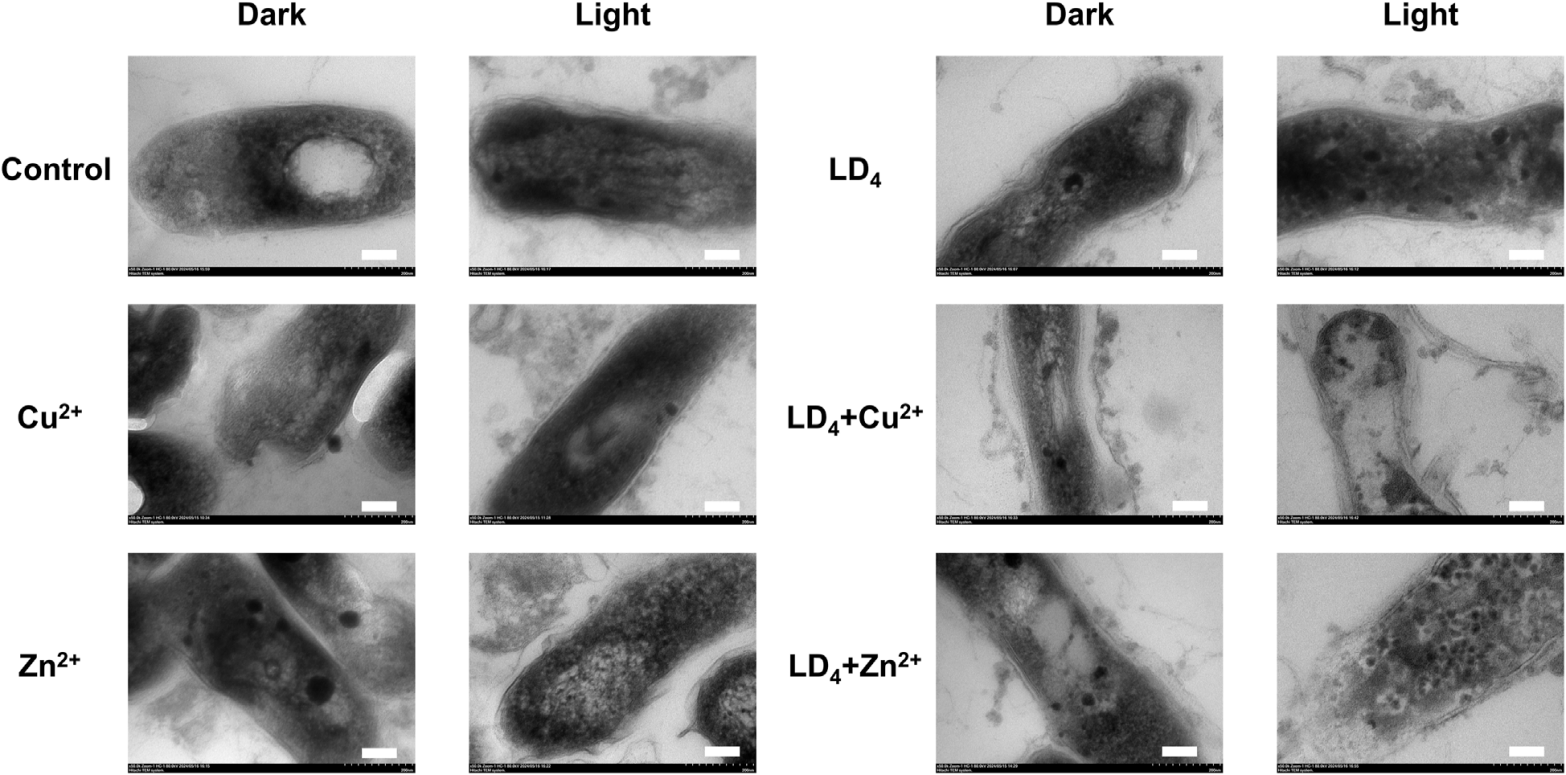
Transmission electron microscopy of H37Rv under the condition of combined use of metal ions and LD_4_. The scale bar represents 100 nm.

TEM analysis was further utilized to explore the mechanism underlying the synergistic antibacterial effect of metal ions and LD_4_. Bacterial morphological changes were examined (Figure 5). The cell wall of *Mtb* contains a high concentration of lipids (Chen et al., 2017). The results demonstrated that H37Rv exhibited intact structural integrity, with a tightly packed cell wall, an undamaged cell membrane, and observable ribosomes in the control group. In contrast, in the Cu^2+^ treatment group, partial indentations were observed in the bacterial cell wall compared to the control group. Additionally, no significant differences were noted between the light and dark condition groups, suggesting that light exposure did not significantly influence the Cu^2+^-induced damage to the *Mtb* cell wall. Bacterial deformation and cell wall indentation were observed in the Zn^2+^ treatment group. However, light exposure did not significantly compromise the structural integrity of the bacteria. In the LD_4_ group, bacterial structures remained largely intact under dark condition, with no clear cell wall indentation or damage. Similarly, under light condition, the bacterial cell wall maintained its integrity. These findings suggest that under light reaction conditions, LD_4_ does not significantly impair the viability of *Mtb*. In the LD_4_ + Cu^2+^ group, detachment of the bacterial cell wall from the cell membrane was observed in the dark reaction group, suggesting that the dark reaction significantly affected the structural integrity of *Mtb* cells. The coordination of Cu^2+^ with the amino groups of LD_4_ formed a copper-ammonia complex with strong oxidizing properties, which induced the separation of the cell wall and cell membrane. Under light condition, the cell wall was severely compromised, leading to leakage of bacterial contents, which severely impaired the physiological activity of *Mtb* and consequently resulted in a strong antibacterial effect. In the LD_4_ + Zn^2+^ group, bacterial structures were relatively intact, and antibacterial activity was weak under dark condition. In contrast, under light condition, the bacterial cell wall exhibited damage, intracellular contents leaked out, and separation of the cell wall from the cell membrane as well as a blank region around the ribosomes were observed. Typically, hydrogen peroxide, peracetic acid, and chlorine dioxide are used as disinfectants for inactivating *Mtb*. However, their effective concentrations are relatively high; for example, hydrogen peroxide requires 0.8%, while peracetic acid requires 0.06%, and these agents must be maintained for 60–90 min to effectively kill *Mtb*. Chlorine dioxide requires an effective concentration of 1–10 g/L (Rutala et al., 1991). Additionally, these three disinfectants exhibit significant cytotoxicity and can cause strong irritation to the respiratory tract.

**FIGURE 6:**
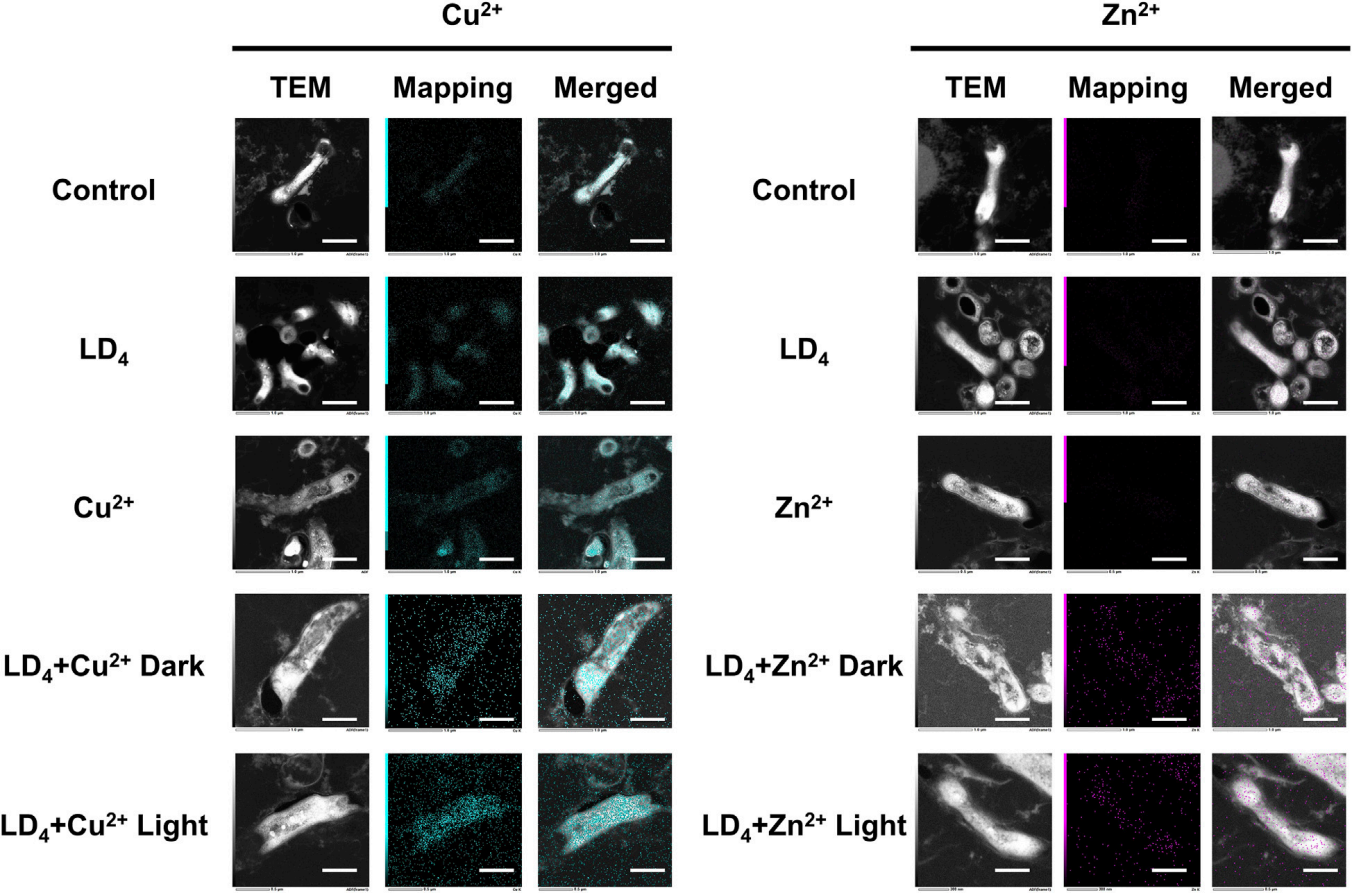
TEM dark-field and mapping results of LD_4_ in combination with Cu^2+^ and Zn^2+^ against *Mtb*. Cu and Zn elements are indicated in blue and purple, respectively. The intensity of mapping signal represents the concentration of the element. The scale bar represents 1 μm. Abbreviations: *Mtb*, *Mycobacterium tuberculosis*; TEM, transmission electron microscopy.

We further quantified the metal ion content within the bacteria using TEM mapping, and the results are presented in Figure 6. Under light condition, the combination of LD_4_ with Cu^2+^/Zn^2+^ induced significant damage to the cell wall of H37Rv, resulting in the leakage of bacterial contents. Mapping analysis of *Mtb* in the combined treatment group revealed that Zn was almost undetectable in the control group, whereas Cu was only present in trace amounts within the bacteria. We observed that in the LD_4_ combined with Cu^2+^/Zn^2+^ group, the Mapping images of Cu and Zn elements showed a high degree of overlap with bacteria cells. Additionally, Cu and Zn were detected in the bacterial efflux contents. These findings suggest that the complexes formed by Cu^2+^/Zn^2+^ and LD_4_ can localize to the bacterial cell wall, cell membrane, and intracellular contents, exerting antibacterial effects via photodynamic action under 650 nm illumination. In the LD_4_ group, a small amount of Cu was observed on the bacterial surface, whereas Zn was scarcely detectable within the bacteria. The results of the Cu^2+^ group showed that there was no significant difference between the bacteria in this concentration group and those in the Control group or the LD_4_ group. This finding may be attributed to the cooperative action of the nutrient copper sensor Mac1p (Graden and Winge, 1997) and the toxic copper sensor Ace1p (Peña et al., 1998), which jointly regulate copper homeostasis in bacteria, ensuring that intracellular copper ions remain within a range that satisfies physiological activity requirements while avoiding toxic accumulation. Mapping results of the Zn^2+^ group demonstrated that intracellular Zn^2+^ levels were extremely low. Zinc homeostasis is primarily regulated by the zinc importer MtNramp1 (Juttukonda and Skaar, 2015) and the zinc efflux protein MtZnT1 in *Mtb* (Dow et al., 2021). MtNramp1 facilitates zinc uptake to fulfill physiological demands, whereas MtZnT1 plays a critical role in preventing cytotoxic levels of intracellular Zn^2+^ accumulation (Tejada-Jiménez et al., 2015). Mapping results from the Cu^2+^ and Zn^2+^ groups demonstrated that *Mtb* exhibited a higher uptake for copper compared to zinc. When LD_4_ was applied alone, the corresponding TEM images revealed a modest increase in copper levels within bacterial cells. In contrast, in the LD_4_ + Cu^2+^ group, where additional Cu^2+^ was supplemented, TEM images indicated a significant increase in intracellular copper ion levels. In the LD_4_ + Zn^2+^ group, the intracellular Zn^2+^ concentration was significantly higher than that in the LD_4_ group, the control group, and the Zn^2+^ group. Previous findings revealed that LD_4_ can form complexes and nanoparticles with Zn^2+^, resulting in a higher singlet oxygen yield compared to LD_4_ alone. Under 650 nm laser irradiation, this enhanced photodynamic antibacterial activity surpassed that of LD_4_ alone. The increased intracellular Zn^2+^ levels in bacteria were found to inhibit the activity of key enzymes, induce oxidative stress, high ROS yield, and interfere with the synthesis of the bacterial cell wall, thereby contributing to its overall antibacterial efficacy. Under high concentration condition, the activities of proteins involved in maintaining Cu^2+^ and Zn^2+^ homeostasis may be inhibited, leading to excessive intracellular accumulation of Cu^2+^ and Zn^2+^, inducing toxicity and, thereby, achieving a greater antibacterial effect.

### 3.6 Cytotoxicity of the combination of metal ions and LD_4_

**FIGURE 7:**
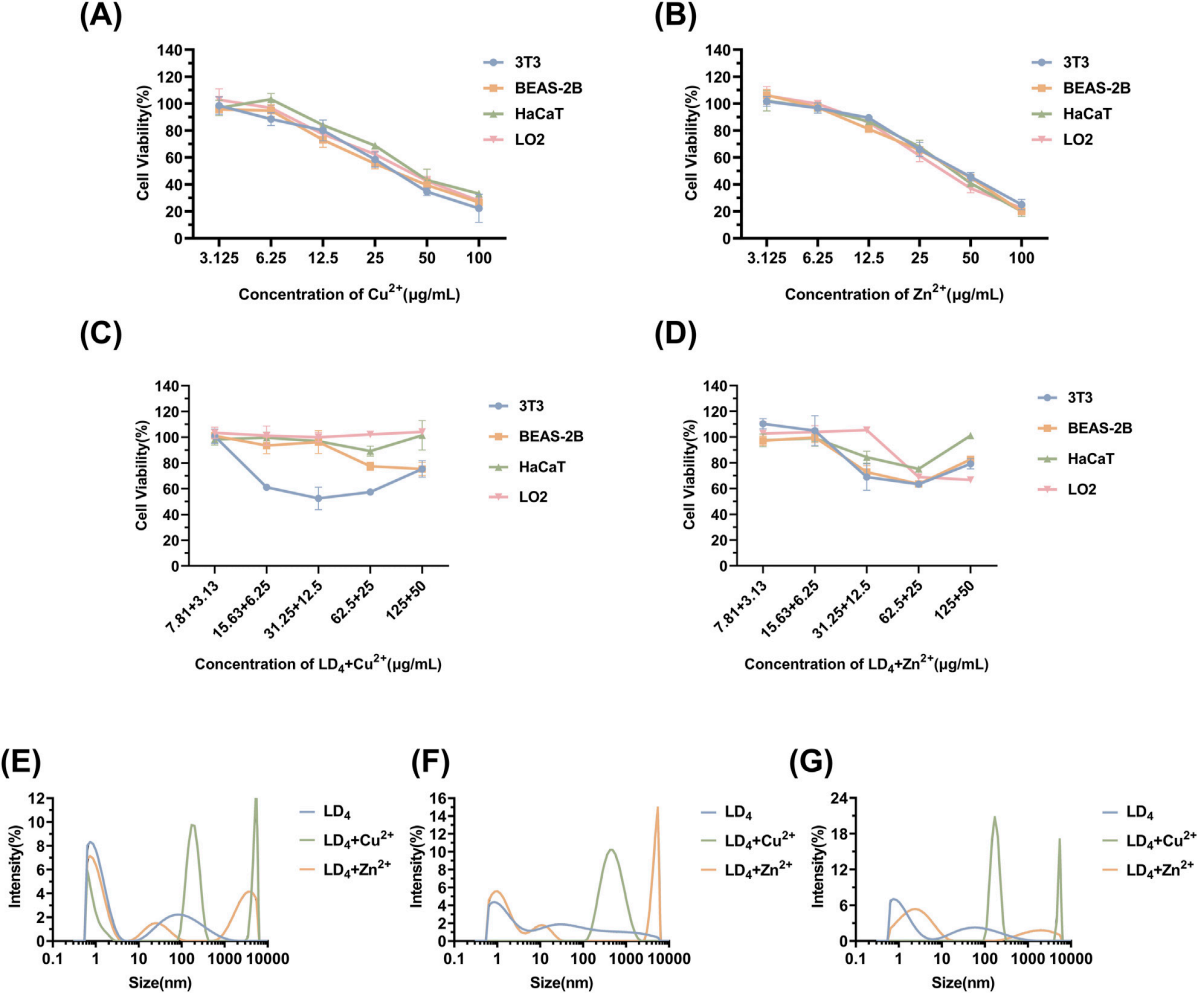
Cytotoxicity of Cu^2+^ **(A)**, Zn^2+^ **(B)**, LD_4_ + Cu^2+^ **(C)**, and LD_4_ + Zn^2+^ **(D)**, as well as the particle size determination results of the system after mixing LD_4_ with Cu^2+^/Zn^2+^ at different concentrations. **(E)** LD_4_ concentration of 31.25 μg/mL and metal ion concentration of 12.5 μg/mL. **(F)** LD_4_ concentration of 62.5 μg/mL and metal ion concentration of 25 μg/mL. **(G)** LD_4_ concentration of 50 μg/mL and metal ion concentration of 50 μg/mL.

Mouse embryonic fibroblast cells (3T3), human immortalized keratinocytes (HaCaT), human normal liver cells (LO2), and human bronchial epithelial cells (BEAS-2B) were selected to evaluate the cytotoxicity under the synergistic cytotoxicity of metal ions and LD_4_ using CCK-8 assays. The results are presented in Figures 7A–D. At low concentrations, the cell survival rate remained relatively high. Under therapeutic dose conditions, the cell viability was maintained >98% across all tested cell lines. However, when the concentration of LD_4_ in combination with Cu^2+^ was gradually increased to 31.25 + 12.5 μg/mL, the survival rate of HaCaT cells decreased significantly to 52.43% ± 8.63%. Notably, at this concentration, no cytotoxic effects were observed in 3T3, BEAS-2B, or LO2 cells, with their survival rates remaining >90%. The survival rate of HaCaT cells gradually increased with the increasing concentration. When the concentration reached 125 + 50 μg/mL, the survival rate increased to 75.36% ± 6.47%. The experimental results for the LD_4_ combined with Zn^2+^ group revealed that HaCaT, 3T3, and BEAS-2B cells exhibited a trend consistent with that observed in the LD_4_ + Cu^2+^ group. Under therapeutic doses, the cell survival rate remained >90%, indicating low cytotoxicity. However, at high concentrations, the cytotoxicity of metal ions combined with LD_4_ was observed. At 62.5 + 25 μg/mL, the viability of HaCaT, 3T3, and BEAS-2B cells was lowest, at 75.13% ± 0.94%, 63.34% ± 1.34%, and 63.48% ± 0.57%, respectively. At 125 + 50 μg/mL, the survival rate of LO2 cells was lowest, at 68.89% ± 7.08%. The possible reason for the initial increase followed by a decrease in cytotoxicity with increasing concentration is that LD_4_ forms dimers or trimers at higher concentrations, thereby reducing metal ion-induced damage to cells and resulting in lower cytotoxicity at high concentrations. To verify the conjecture, the changes in particle size under various concentration combinations were determined.

The results of particle size determination are presented in Figures 7E–G. Upon mixing the LD_4_ aqueous solution with the metal ion aqueous solution, significant aggregation was observed, with a significant increase in the proportion of particles measuring >1 μm and an overall rise in average particle size. In contrast to the observations, the LD_4_ aqueous solution exhibited less particle aggregation. Notably, most of the particulate matter was retained in a dissolved colloidal state throughout the experimental duration, as evidenced by dynamic light scattering analysis. The cytotoxicity trend observed in Figures 7C,D may be attributed to the formation of polymers through the combination of some LD_4_ molecules with metal ions. This process complexes the toxic metal ions, thereby reducing their cytotoxic effects and ultimately enhancing cell survival rates.

## 4 Conclusion

In this study, we designed and validated a synergistic antibacterial strategy by combining metal ions with LD_4_. Our findings revealed that both Cu^2+^ + LD_4_ and Zn^2+^ + LD_4_ demonstrated significant synergistic antibacterial effects against *C. albicans* and *Mtb*. The synergy was quantified using FICI values, ranging 0.0625–0.281, confirming a strong synergistic interaction. The mechanistic investigation revealed the distinct antibacterial pathways of LD_4_ + Cu^2+^ and LD_4_+Zn^2+^. Specifically, the combination of Zn^2+^ with LD_4_ led to a significant increase in ROS yield, reaching 322.35% ± 7.30% of that recorded after use of LD_4_ alone. In contrast, Cu^2+^ induced fluorescence quenching of LD_4_, which resulted in a relatively lower ROS yield. The LD_4_ + Cu^2+^ group demonstrated stronger antibacterial activity, primarily due to the physical disruption of the lipid-rich cell wall. This finding was further substantiated by TEM images showing cell wall separation and cytoplasmic leakage under dark condition. Fluorescence and infrared spectroscopy analyses demonstrated that metal coordination modified the fluorescence state of LD_4_ and elucidated the changes in functional groups upon complexation with metal ions. Furthermore, a systematic investigation was conducted into the intrinsic molecular mechanisms underlying the variations in fluorescence intensity. Although the antibacterial efficacy was significant, under therapeutic doses, the survival rate of normal cells remained >90%. Cytotoxicity becomes apparent only when the concentration exceeds the therapeutic range. Systematic analysis of particle size changes in both LD_4_ + Cu^2+^ and LD_4_ + Zn^2+^ combination groups revealed the concentration-dependent cytotoxicity anomaly, where elevated drug concentrations initially intensified cytotoxic effects before subsequent attenuation at higher dosage levels. In conclusion, this study elucidated the synergistic antibacterial mechanisms of metal ions (Cu^2+^ and Zn^2+^) with LD_4_, confirmed their significant potential in antibacterial therapy, and presented a promising strategy for combating fungal and mycobacterial infections.

## Data Availability

The original contributions presented in the study are included in the article/Supplementary Material, further inquiries can be directed to the corresponding author.
